# Carbon-Encapsulated Co_3_O_4_ Nanoparticles as Anode Materials with Super Lithium Storage Performance

**DOI:** 10.1038/srep16629

**Published:** 2015-11-13

**Authors:** Xuning Leng, Sufeng Wei, Zhonghao Jiang, Jianshe Lian, Guoyong Wang, Qing Jiang

**Affiliations:** 1 Key Laboratory of Automobile Materials, Department of Materials Science and Engineering, Jilin University, No. 5988 Renmin Street, Changchun 130025, PR China; 2 Key Laboratory of Advanced Structural Materials, Changchun University of Technology, No.2055 Yanan Street, Changchun 130012, PR China

## Abstract

A high-performance anode material for lithium storage was successfully synthesized by glucose as carbon source and cobalt nitrate as Co_3_O_4_ precursor with the assistance of sodium chloride surface as a template to reduce the carbon sheet thickness. Ultrafine Co_3_O_4_ nanoparticles were homogeneously embedded in ultrathin porous graphitic carbon in this material. The carbon sheets, which have large specific surface area, high electronic conductivity, and outstanding mechanical flexibility, are very effective to keep the stability of Co_3_O_4_ nanoparticales which has a large capacity. As a consequence, a very high reversible capacity of up to 1413 mA h g^−1^ at a current density of 0.1 A g^−1^ after 100 cycles, a high rate capability (845, 560, 461 and 345 mA h g^−1^ at 5, 10, 15 and 20 C, respectively, 1 C = 1 A g^−1^), and a superior cycling performance at an ultrahigh rate (760 mA h g^−1^ at 5 C after 1000 cycles) are achieved by this lithium-ion-battery anode material.

Lithium-ion batteries (LIBs), as a kind of electrochemical energy conversion and storage device, have been intensively explored during the past decades for the fact that they have been widely applied as power sources for portable electronic devices^1^,^2^. Carbon-based materials are commonly used as an anode for LIBs^3^. But low theoretical capacity (372 mA h g^−1^) is the major concern, which puts sand on the wheel of its application in hybrid electric vehicles and electric vehicles where a power source with high energy density and high power density is needed^1^,^4^. Thus state-of-the-art LIBs can be explored only when eximious alternative electrode materials with high capacity, high conductivity, super rate capability and long cycle life are found.

Transition metal oxides (M_x_O_y_: M = Co, Fe, Ni, Cu, Mn, etc.)^5^,^6^,^7^,^8^,^9^,^10^,^11^,^12^,^13^ are considered as feasible anode alternatives because of their high specific capacities induced by conversion reaction mechanism, which was first proposed and elucidated by Poizot *et al.*^14^. However, bulk transition metal oxides are still far from application as LIB anode materials because they suffer from problems associated with the relatively poor electrical conductivity and the large volume change that occurs during the Li^+^ insertion and extraction^15^. Poor conductivity always leading to low power density and large volume change will induce pulverization^16^, which result in an insufficient lithium storage performance and a rapid capacity decay^17^. Thus, fabrication of high-performance transition metal oxide electrode materials with large reversible capacities and good cycling stabilities remains a great challenge.

Currently a popular strategy toward alleviating these problems is nanostructuring. Compared to their bulk counterparts, nanosized electrode materials own many merits. Firstly, nanosized materials are generally too tiny to be pulverized^18^,^19^. Meanwhile the void between the nanosized electrode materials can provide spaces to buffer the mechanical stress induced by the volume change^17^. Consequently, nanostructured electrode materials usually have a higher reversible capacity as well as a longer cycle life than their bulk counterparts. Secondly, nanostructured electrode materials hold a very high surface-to-volume ration, which provide plenty of reaction sites and shorter diffusion paths^20^. Thus, these electrodes present a high rate capacity and a high power density. Substantial efforts have been made on nanostructuring and huge progress has been made on it^4^,^21^,^22^. Meanwhile, these works also obviously show that nanostructuring can’t solely solve all the problems thoroughly that transition metal oxides have suffered as LIBs anode materials. Nanoparticles (NPs) tend to aggregation after tens of cycling. This problem has been partially alleviated by separating them by graphene^6^,^23^,^24^ which is a new two-dimensional carbon material owning superior electrical conductivity, high surface-to-volume ration, ultrathin thickness, structural flexibility, and chemical stability^25^. But such physical adsorption is not very strong enough. After dozens of circulations, they can also be desorbed from the surface. The nanovoids included in the nanostructured electrode materials can’t strongly support the structural stability and integrity. The initial morphology generally loses after dozen of cycles because of pulverization. If these nanovoids can be filled in by some solid materials which not only give a support to the nanostructued materials and buffer the inner stress but also improve the electric conductivity, then the LIBs anode materials are integrated as a unite. The properties of LIBs anode must be meliorated a lot.

Cobaltosic oxide (Co_3_O_4_) has received much attention in LIBs on the basis that it has a high theoretical capacity of 890 mA h g^−1^, more than twofold of the currently commercial graphite^26^,^27^. In order to overcome the shortage of bulk Co_3_O_4_ electrode, different kind of nanostructured Co_3_O_4_ combining various forms of carbon were designed as LIBs anode materials, which really improved their electrochemical performance^3^,^16^,^25^,^26^,^28^,^29^,^30^,^31^,^32^,^33^,^34^,^35^,^36^. In this paper, the synthesized Co_3_O_4_ NPs with an average grain diameter of 20.4 nm were successfully encapsulated by a layer of thin carbon shell, forming Co_3_O_4_@C core-shell structures which were homogeneously embedded in porous graphitic carbon (designed as Co_3_O_4_@C@PGC) nanosheets. The electrochemical properties of such materials were tested as a LIBs anode. It presented very high reversible capacity and excellent rate performance and cycling stability. It might be possible to extend this technique to other kind of nanostructured matal oxide for important applications in high-performance LIBs, supercapacitors, adsorbents, catalysts and sensors in many scientific disciplines.

## Results and Discussion

**Figure 1:**
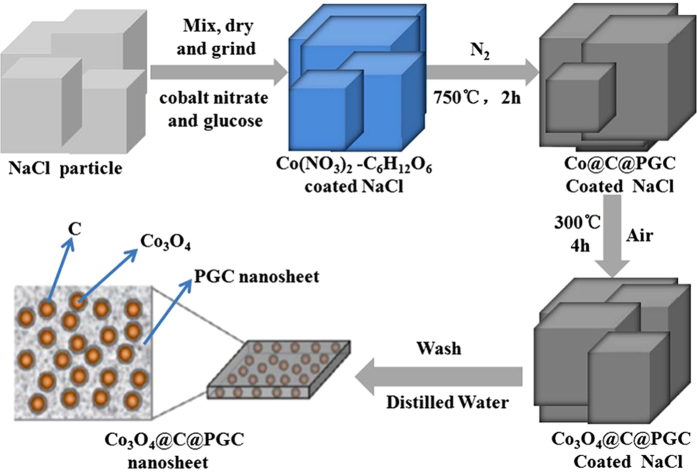
Schematic illustration of the *in situ* sol-gel technique to fabricate Co_3_O_4_@C@PGC nanosheets by using the surface of NaCl particles as the template.

The overall synthetic procedure of the Co_3_O_4_@C@PGC nanosheets is schematically illustrated in Fig. 1. Two strategies were involved in this procedure. Firstly, the water-soluble NaCl particle surface was utilized as the template for the nanosheets because NaCl has a face-centered cubic (fcc, Fm3m (225), a = 0.5642 nm) crystal structure, and its growth rate can be controlled by varying the concentration and temperature^37^,^38^,^39^. Secondly, glucose and cobalt nitrate were chosen as the carbon precursor and the metal precursor, respectively, due to their low cost. In the synthesis, the NaCl, C_6_H_12_O_6_ and Co(NO_3_)_2_ were first dissolved in distilled water to get a homogeneous solution. After gradually heated to 80 °C, the solution color changed from pink to blue, the sample viscosity increased greatly, and then the polymerization of the glucose began, which resulted in the generation of a very thin frame homogeneously coated on the NaCl particle surface. After that, the composite powders were calcinated at 750 °C under N_2_. During this process, the Co(NO_3_)_2_ should be decomposed into cobalt oxide^40^. The oxide was subsequently reduced by C_6_H_12_O_6_ into Co NPs with the protection of inactive gas^29^,^35^. The fresh generated carbon layer usually encapsulated the Co NPs firstly, and then piled up in the form of porous graphitic carbon. As a result, the coating layer on the surface of the NaCl particles was converted to carbon-encapsulated Co NPs embedded in porous graphitic carbon (designed as Co@C@PGC) nanosheets. In the second step, the Co NPs were further *in situ* oxidized into Co_3_O_4_ NPs as the Co@C@PGC nanosheets were calcinated in air. Finally, the Co_3_O_4_@C@PGC nanosheets were obtained on the surface of the NaCl particles as shown by the images of Fig. S1.

**Figure 2:**
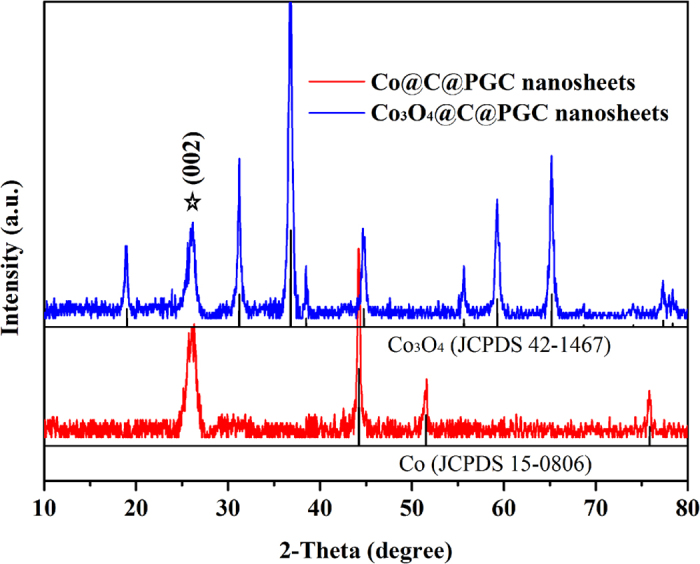
The typical XRD patterns of the Co@C@PGC nanosheets and Co_3_O_4_@C@PGC nanosheets.

The XRD patterns of Co@C@PGC and Co_3_O_4_@C@PGC nanosheets were both shown in Fig. 2. A peak appears at 2θ=26.5° in both XRD patterns, which corresponds to the diffraction peak of (002) crystallographic plane of graphite (JCPDS 65-6212). That means the carbonization of C_6_H_12_O_6_ takes place at step one, and the annealing in air at step two has little damage on the carbon. Except of graphite, all peaks in the XRD pattern of Co@C@PGC belong to Co (JCPDS 15-0806), which implies that the cobalt has been completely reduced from its oxide. Only Co_3_O_4_ phase is shown in the XRD pattern after calcinations in air except of graphite. It suggests that the calcination in step two is very effective to accomplish the conversion from the precursor (Co@C@PGC nanosheets) to the targeted sample (Co_3_O_4_@C@PGC nanosheets). The average sizes of NPs were both calculated based on the full width at half maximum intensity of (311) for Co_3_O_4_ and (111) for Co using Scherrer’s formula. The estimated average particle size of Co_3_O_4_ is very close to Co, which is about 20.4 nm. In addition, NaCl phase isn’t found in both XRD patterns. So NaCl particles were completely removed in the water-washing process.

**Figure 3:**
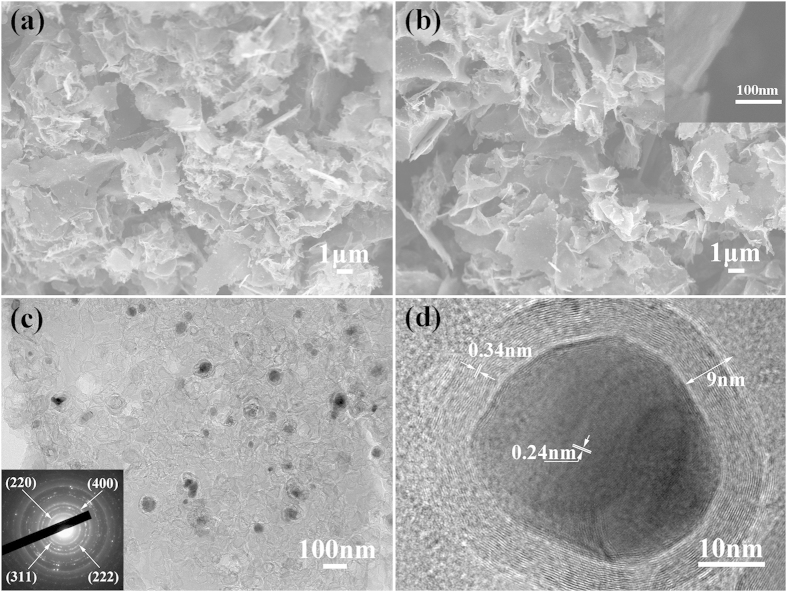
(**a**) SEM image of Co@C@PGC nanosheets; (**b**) SEM image of Co_3_O_4_@C@PGC nanosheets and the inset is SEM image of high magnification; (**c**) TEM and (**d**) HRTEM image of Co_3_O_4_@C@PGC nanosheets.

The morphology of Co@C@PGC and Co_3_O_4_@C@PGC nanosheets was shown in the SEM image of Fig. 3a,b, respectively. As shown in Fig. 3a, a lot of nanosheets with a thickness of about 20 nm and an area of 1–10 μm^2^ interconnected with each other to build up a porous architecture. This architecture can be reserved even after calcinations in air, which is evidenced by the SEM image shown in Fig. 3b. The inset in Fig. 3b clearly shows that the thickness of the nanosheets is around 20 nm. The morphology of Co_3_O_4_/C composites without adding NaCl was also observed by SEM, shown in Fig. S2. The flakes are very thicker and larger than Co_3_O_4_@C@PGC nanosheets. Their thickness is more than 500 nm. They just pile up on each other, leaving very little space between them. The thin sheets observed in Co@C@PGC and Co_3_O_4_@C@PGC disappear thoroughly in Co_3_O_4_/C composites. It suggests that NaCl particles have played a crucial role in building up such porous architecture of nanosheets. A TEM image and HRTEM image of Co_3_O_4_@C@PGC nanosheets are shown in Fig. 3c,d, respectively. The graphite carbon nanosheets seem to be transparent to the electron beam and show a very low contrast in the image, which again suggests that the nanosheet is very thin, around tens of nanometers. The black rings located on the sheet of Fig. 3c indicate a foam-like structure of the graphite. Co_3_O_4_ NPs, with diameters ranging from 5 to 40 nm, are well-dispersed in the PGC nanosheet. The selected area electron diffraction pattern in the inset of Fig. 3c further verifies the encapsulated core being Co_3_O_4_ NPs. In fact, the Co_3_O_4_ NPs were firmly anchored by the porous graphite nanosheets. As shown in Fig. 3d,a Co_3_O_4_ crystal is perfectly encapsulated by thin and well-graphitized onion-like carbon shells. The HRTEM interference fringes of the Co_3_O_4_ crystal can be clearly decerned. They have a spacing of 0.24 nm, as denoted in Fig. 3d, which is equal to the spacing of (311) plane of Co_3_O_4_. The spacing of the interference fringe of the shell is 0.34 nm, which is also equal to the spacing of (002) plane of graphite. And the thickness of graphite shell is about 9 nm. The morphology of Co@C@PGC nanosheets is also characterized by TEM and HRTEM (Fig. S3a,b). Carefully comparing the results, it is found that there is little difference on the morphology of the graphite carbon sheets and the crystal sizes. It again makes sure that the calcinations didn’t hurt the graphite carbon sheets and none coalescence happened among Co NPs. The Co NPs just were oxidized at its place and converted to Co_3_O_4_ NPs.

**Figure 4:**
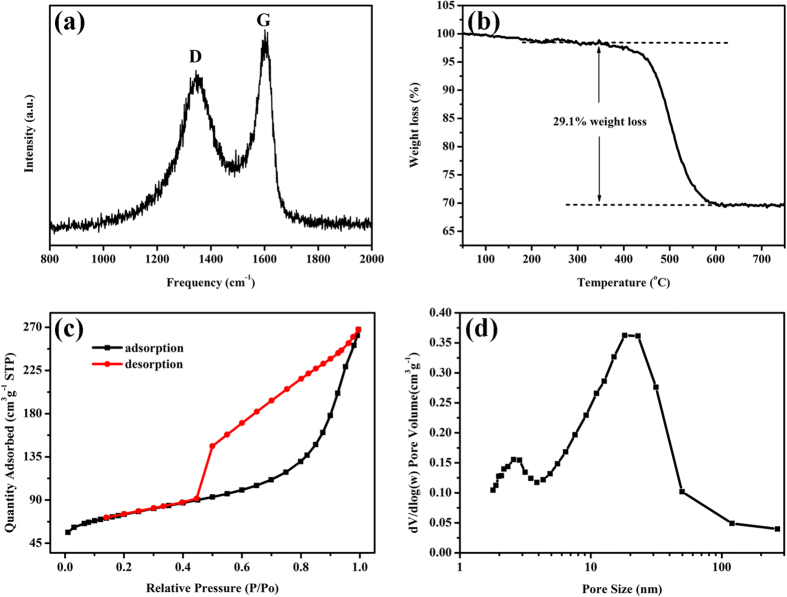
(**a**) Raman spectrum of the Co_3_O_4_@C@PGC nanosheets; (**b**) The TGA profile of the Co_3_O_4_@C@PGC nanosheets; (**c**) The N_2_ adsorption/desorption isotherms and (**d**) the pore size distribution of the Co_3_O_4_@C@PGC nanosheets.

The Raman spectrum shown in Fig. 4a further confirms the existence of graphitic structure in Co_3_O_4_@C@PGC nanosheets. Both G band and D band obviously appeared the spectrum at 1603 cm^−1^ and 1346 cm^−1^, respectively. The G band is the result of a radial C=C stretching mode of sp^2^-bonded carbon in the basal plane of the crystalline graphite. Thus it is considered as the intrinsic Raman signal for graphite. The D band is associated with disorder, allowing zone edge modes of the graphite structure to become active due to the lack of long-range order in amorphous and quasi-crystalline forms of carbon materials^41^,^42^. Thus, the integral intensity ratio of G peak to D peak (I_G_/I_D_) is usually used to evaluate the degree of crystallization of carbon materials^43^. The value of I_G_/I_D_ for the Co_3_O_4_@C@PGC nanosheets is ~1.3, indicating that the obtained nanosheets own well-crystallized graphitic structures^44^. This will be beneficial for electronic conduction of Co_3_O_4_ NPs. The carbon content is determined by TGA and the result is shown in Fig. 4b. As the sample is heated to 750 ^o^C in air, the carbon will be completely burnt out. So, the weight loss in TGA result is equal to the carbon content. The carbon content of the Co_3_O_4_@C@PGC nanosheets is 29.1 wt% which is comparable to the Co_3_O_4_/C composites (30.0 wt%, Fig. S4).

Figure 4c,d show the N_2_ adsorption/desorption isotherms and pore size distributions of the Co_3_O_4_@C@PGC nanosheets, respectively. The plot shown in Fig. 4c is a typical type IV N_2_ adsorption−desorption isotherm^45^ with a distinct hysteresis loop in the relative pressure range of 0.45−0.97, indicating a mesoporous structure and a narrow mesopore size distribution. In addition, the steep slope above 0.97 and the slow slope below 0.45 indicate the existence of macropores and micropores in the nanosheets. The pore size distribution was plotted in Fig. 4d. The pore size distribution ranges from 1.7 to 300 nm and has two preponderances at 2.5−2.8 nm and 18−22 nm. The average pore diameter is about 9.2 nm based on Barrett–Joyner–Halenda (BJH) adsorption. The BET specific surface area is measured to be ~246 m^2^ g^−1^. These results particularly characterize the porous structure of the graphite carbon sheets. Obviously, such small crystal size of Co_3_O_4_, which is comparable to the pore size, is incapable to contribute more pore and surface area. The porous structures of Co_3_O_4_@C@PGC nanosheets are vital for the electrochemical performance because they provide easy diffusion for electrolyte and facile accessibility of the active sites. They are also effective to accommodate huge volume change during lithium ion insertion/extraction and maintain the integrity, increase the electronic conductivity, and promote the formation of uniform SEI films and improve the stability of them^46^,^47^, which endows the LIBs excellent cycle stability.

**Figure 5:**
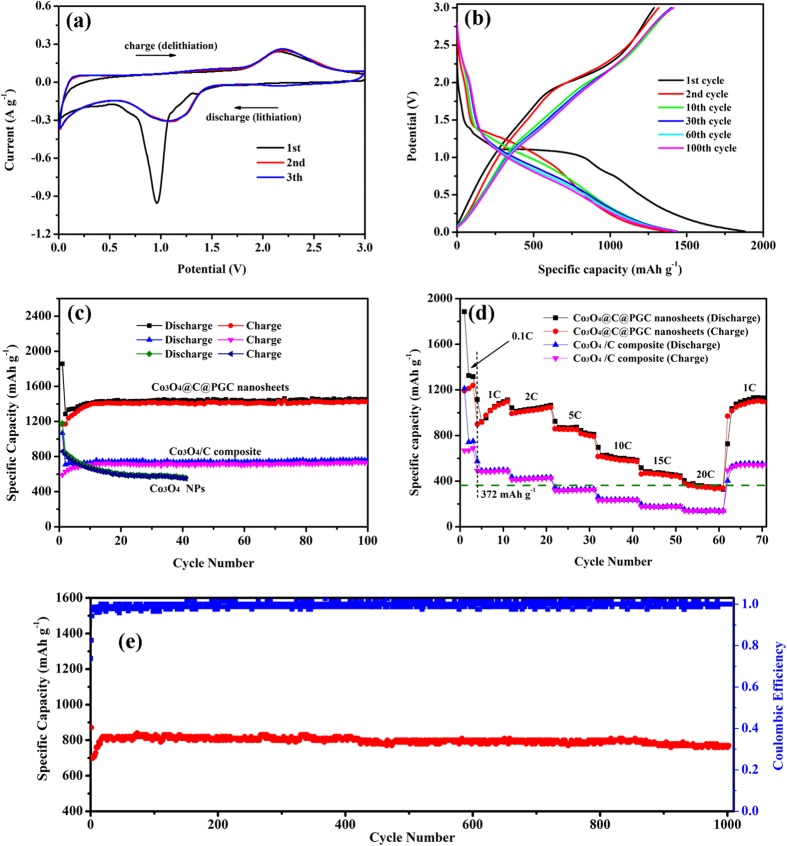
(**a**) Representative CV curves of an electrode based on the Co_3_O_4_@C@PGC nanosheets obtained at a voltage range of 0.0 to 3.0 V (vs Li^+^/Li) and potential scan rate of 0.1 mV s^−1^. (**b**) Voltage profiles of the Co_3_O_4_@C@PGC nanosheets electrode at a current density of 0.1 C. (**c**) Charge/discharge capacities of the Co_3_O_4_@C@PGC nanosheets, Co_3_O_4_/C composite and Co_3_O_4_ NPs at a current density of 0.1 C. (**d**) Rate capabilities and cycle performance of Co_3_O_4_@C@PGC nanosheets and Co_3_O_4_/C composite electrodes cycled at different rates from 0.1 to 20 C. (**e**) Cycle performance and Coulombic efficiency for the Co_3_O_4_@C@PGC nanosheets electrode at a higher current density of 5 C. (1 C = 1 A g^−1^).

The initial three cycles of the representative cyclic voltammogram (CV) performed on a coin half-cell at room temperature between 0.01 and 3.0 V at a sweep rate of 0.1 mV s^−1^ is presented in Fig. 5a. In accord with previous results, the first cycle shows lots of differences from the subsequent ones on CV curves, especially on the discharge branch^21^,^48^,^49^. In the first discharge process, the obvious cathodic peak was located at around 0.97 V, which was attributed to the electrochemical reduction of Co_3_O_4_ to metallic cobalt accompanying the formation of Li_2_O and the solid electrolyte interphase (SEI) film^26^,^28^. In the anodic process, broad peaks located at around 2.15 V can be ascribed to the reversible oxidation reaction from cobalt to Co_3_O_4_^23^. The total electrochemical reaction mechanism of Co_3_O_4_ anode can be described by the following electrochemical conversion reaction^50^:


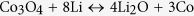


The main reduction peak is shifted to ~1.15 V, and the peak intensity drops significantly from the second cycle, indicating the occurrence of some irreversible reactions associated with formation of the SEI film in the first cycle^13^. On the other hand, the oxidation peak at ~2.2 V in the anodic sweep exhibits little change during the three cycles, suggesting that the SEI formed on the PGC nanosheets surfaces in the first cycle is very stable.

Typical galvanostatic charge/discharge curves of the Co_3_O_4_@C@PGC nanosheets electrodes at a current density of 0.1 A g^−1^ between 0.01 and 3.00 V are shown in Fig. 5b. The initial charge and discharge capacities are approximately 1187 and 1859 mA h g^−1^, respectively, resulting in an initial Coulombic efficiency of ~64%. The initial irreversible capacity loss of the Co_3_O_4_@C@PGC nanosheets could be associated with the inevitable formation of SEI and decomposition of electrolyte^51^,^52^ and in good agreement with the above CV results. The discharge voltage plateau at ~1.1 V in the first cycle shift to ~1.2 V since the second cycle, just corresponding to the shift of reduce peak in CV curves from 0.97 V to 1.15 V. The areas under the charge and discharge curves are comparable, indicating a very low energy loss during charge/discharge. Starting from the 10th cycle, both charge and discharge curves are overlapping the previous one up to the 100th cycle, which indicates that the Co_3_O_4_@C@PGC nanosheets exhibit excellent cycle stability^24^,^53^.

The cycle performances of the Co_3_O_4_@C@PGC nanosheets, Co_3_O_4_/C composite and Co_3_O_4_ NPs were investigated at a current density of 0.1 A g^−1^. The results were shown in Fig. 5c. The Co_3_O_4_@C@PGC nanosheets electrode has the best cyclic retention and the highest reversible capacity. After 100 cycles, the reversible capacity is as high as 1413 mA h g^−1^. The capacity is even higher than the theoretical capacity of Co_3_O_4_, which is common in many reported literatures^19^,^26^,^28^,^29^,^54^,^55^,^56^,^57^. Such high specific capacity should be related to the following points: firstly, the tiny Co_3_O_4_ NPs have large specific surface area where more active sites for lithium ions are located^19^. Secondly, the reversible decomposition of the electrolyte and extra lithium ion adsorption/desorption on the SEI during cycling may lead to the high experimental lithium storage capacity as well^27^,^58^. Thirdly, the pure PGC nanosheets have a reversible capacity of 580 mA h g^−1^ (Fig. S5), so there would be synergetic effects between graphitic carbon and Co_3_O_4_ NPs^26^,^55^. In contrast, the reversible capacity of the Co_3_O_4_/C composite is much lower, which is only ~763 mA h g^−1^ at the end of the 100 cycles. But it also has an excellent cycling stability. Although, the bulk carbon in Co_3_O_4_/C composite prevents the Co_3_O_4_ NPs from playing their best in the lithium storage capacity, it still takes an important role in holding the integrality of the structure. The Co_3_O_4_ NPs show the fastest capacity fading and the lowest reversible capacity which is below 549 mA h g^−1^ after 40 cycles. In the case of bare Co_3_O_4_ NPs, SEI trends towards rupture to accommodate the volume change induced by Li^+^ expansion/contraction. The Co NPs forming in discharging processes also have catalyzing effect on SEI and are harmful to the integrality of the film. Thus the electrode material surface will cyclically expose itself to the electrolyte and more and more SEI films consecutively form on it, which leads to continual consuming of electrolyte and the fast capacity fading^3^,^59^. However, a very thin carbon shell covers the electrode material in Co_3_O_4_@C@PGC nanosheets, which induces a strong synergistic effect between porous graphitic carbon nanosheets and carbon-encapsulated Co_3_O_4_ NPs^26^. During the charge/discharge process, the carbon shell is very beneficial to stabilize SEI film and avoid rupturing. Meanwhile, it is also effective to prevent the Co_3_O_4_ NPs from agglomerating. The porous sheet structure of carbon which links the shelled Co_3_O_4_ NPs provides enough space to accommodate the huge volume changes. In addition, the high specific surface area of Co_3_O_4_@C@PGC nanosheets ensures a high contact area between the electrode and electrolyte. So Li^+^ has plentiful diffusion accesses from electrolyte into electrode interior, which will intensively enhance the transport rate of Li^+^. All these factors are vital for the electrode to retain the excellent cycling stability and high reversible storage capability^11^.

The electrode made by Co_3_O_4_@C@PGC nanosheets also has the best rate capability, as shown in Fig. 5d. After three cycles at 0.1 C, the electrodes were firstly tested at 1 C (1 C = 1 A g^−1^). It is obvious that the Co_3_O_4_@C@PGC electrode still needs ten more cycles to activate all active materials for its reversible capacity increases with cycle number until the 11th cycle^50^,^56^. Meanwhile, the Co_3_O_4_@C composite electrode shows a stable capacity as tested at 1 C. The reversible capacity of the Co_3_O_4_@C@PGC nanosheets electrode reaches a very high value of 1105 mA h g^−1^ at the eleventh cycle^23^,^60^, while the Co_3_O_4_@C composite electrode maintains a reversible capacity of 502 mA h g^−1^ during these cycles. At each tested specific current density including 2, 5, 10, and 15 C, the average reversible capacity of the Co_3_O_4_@C@PGC electrode (1030, 845, 560 and 461 mA h g^−1^) is much higher than Co_3_O_4_@C composite electrode (430, 323, 240, 185 mA h g^−1^). Even at 20 C, the average reversible capacity (345 mA h g^−1^) is still very close to the theoretical capacity of graphite. When the current rate finally returns to the initial value of 1 C after 61 cycles, the electrode can recover its initial capacity of 1030 mA h g^−1^ with a little bit loss. And it is still sustainable up to the 70th cycle. The high reversible capacity and excellent rate performance indicate that the electrode has a high energy density and power density. So the electrode occupies the up-right corner of Ragone plot, as shown in Fig. S6^61^. In order to further confirm the durability of this nanosheet anode to work at a higher rate, the cyclability of the Co_3_O_4_@C@PGC nanosheets electrode has been further investigated upon 1000 cycles at 5 C and the result is shown in Fig. 5e. In the initial 20 cycles, the specific capacity gradually increases up to 820 mA h g^−1^, corresponding to a process of activating the active substance, which is common in the literature^50^,^56^. It is striking to note that the specific capacity can remain as high as 760 mA h g^−1^ even after 1000 cycles.

**Figure 6:**
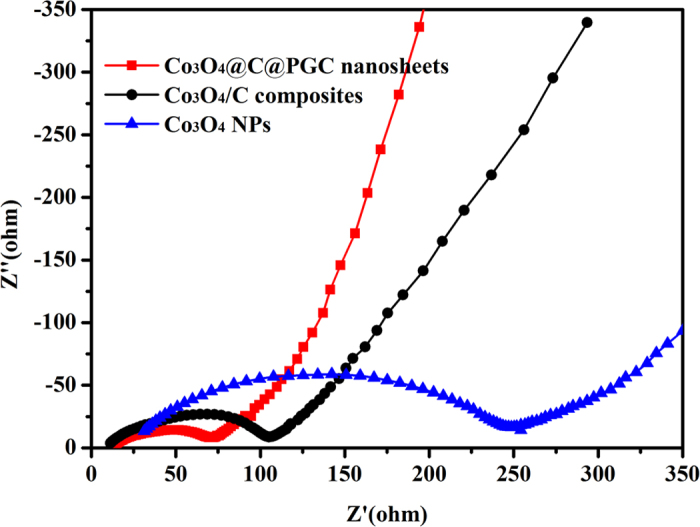
Nyquist plots of the Co_3_O_4_@C@PGC nanosheets, Co_3_O_4_/C composite and Co_3_O_4_ NPs after 10 cycles at a current density of 0.1 C over the frequency range from 100 kHz to 0.01 Hz.

In order to uncover the mechanism which endow the electrode with superior electrochemical performance, electrochemical impedance spectroscopy (EIS), a promising tool for investigating diffusion issues, was conducted at frequencies from 100 kHz to 0.01 Hz on the electrode of Co_3_O_4_@C@PGC nanosheets, Co_3_O_4_/C composite, and Co_3_O_4_ NPs after 10 cycles at a current density of 0.1 C, respectively. The corresponding Nyquist plots are shown in Fig. 6, which is vital to clarify the electrode kinetics. The three impedance spectra have similar features: a medium-to-high-frequency depressed semicircle and a low-frequency linear tail. The semicircle at high frequency is an indication of SEI resistance (R_SEI_) and contact resistance (R_f_), the semicircle across the medium-frequency region represents the charge-transfer impedance (R_ct_) on the electrode/electrolyte interface, and the low-frequency linear tail corresponds to the Warburg impedance (Z_w_) associated with the diffusion of lithium ions in the bulk electrode (R_e_)^7^,^36^,^44^,^62^,^63^. The Co_3_O_4_@C@PGC nanosheets electrode has the smallest semicircle diameter. So it possesses the lowest contact and charge-transfer impedances. That is to say, Li^+^ and electron are able to transport fastest during the electrochemical insertion/extraction reaction in the electrode, which makes it reasonable that the Co_3_O_4_@C@PGC nanosheets electrode shows the best rate performance.

**Figure 7:**
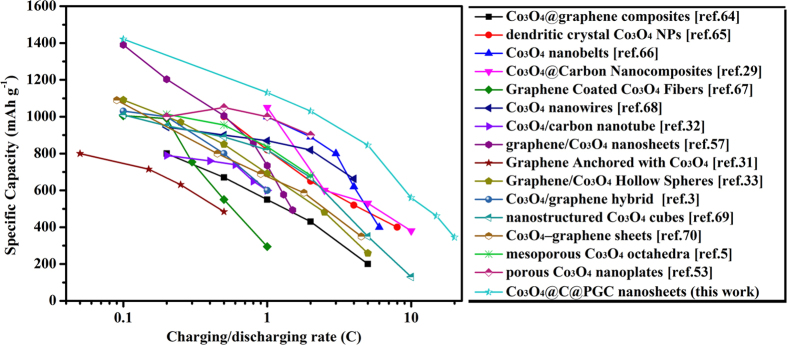
Comparison of capacity at different rates for Co_3_O_4_@C@PGC nanosheets electrode with those of Co_3_O_4_ nanostructure and Co_3_O_4_/C composite anodes reported.

**Figure 8:**
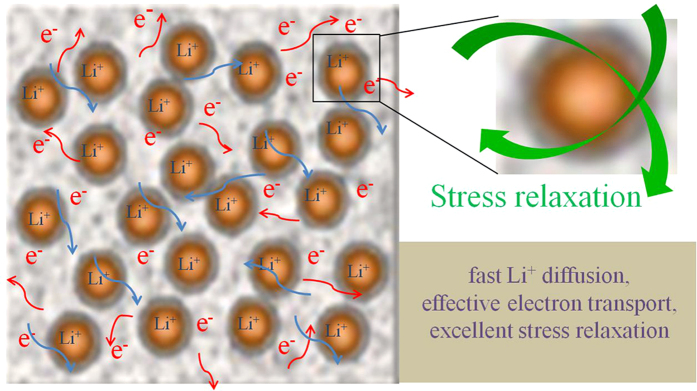
Schematic illustration of Co_3_O_4_@C@PGC nanosheets with fast Li^+^ diffusion, effective electron transport, and excellent stress relaxation during Li^+^ extraction/insertion.

As compared with the results in literature^3^,^29^,^30^,^31^,^32^,^33^,^57^,^64^,^65^,^66^,^67^,^68^,^69^,^70^, the Co_3_O_4_@C@PGC electrode possesses a much larger reversible capacity at any rate, as shown in Fig. 7. This should be related to the unique structure it has. The synthesis techniques and structure characters were summarized in Table S1. The excellent electrochemical performance of Co_3_O_4_@C@PGC nanosheets anode can be illustrated by the mechanism suggested in Fig. 8 and Fig. S7. The size of the Co_3_O_4_ NPs in the paper is small enough. It is very close to the minimal size below which pulverization can’t happen^18^,^19^. Meanwhile the Co_3_O_4_ cores are firmly covered by well-graphitized onion-like carbon shells. Either the pulverization wouldn’t happen in this experiment, or, even if it happened, the particle would be split into finite particles which still connect with the carbon shell, just as illustrated by Fig. S7. Thus it doesn’t cause any loss in lithium storage. Because the NPs are firmly anchored by the carbon shell, it can’t migrate and coalescent with adjacent particles. Thus aggregation of NPs is eliminated in this material. Co nanocrystals would be generated by Co_3_O_4_ NPs because of the reversible conversion reaction^19^. They are very harmful to SEI films, because the fresh Co surface has strong catalytic power on the decomposition of SEI films. But in this experiment, the Co crystals are completely separated by carbon shell. The SEI film should be more stable than the literature. Carbon materials always have good mechanical flexibility, which endow the shells with the ability to expand and contract with Co_3_O_4_ NPs during lithium ion insertion/extraction process. Finally, the volume change is accommodated by the abundant macropore and mesopore in the PGC nanosheets. So the structure of the material is so robust which guarantee the stability of the reversible capacity during cycle charge/discharge. The Co_3_O_4_ NPs were integrated into PGC nanosheets. Thus, a continuous network is constructed by the conductive carbon, which ensures the electron transport freely. The porous structure of the nanosheets provides a huge interfacial area with electrolyte. The electrode has abundant accesses for Li^+^ to interior active materials. The thin film structure of Co_3_O_4_@C@PGC nanosheets shortens the diffusion path of Li^+^. So the electrode can work very well at a very high specific current density. At last, the thin film structure is also very favorable for the adsorption of lithium ions on both sides, edges, and other defects of these nanosheets^59^,^62^. In addition, there may be a strong synergistic effect between PGC nonasheets and Co_3_O_4_ NPs^26^. That may explain the reason why the specific capacity of the Co_3_O_4_@C@PGC electrode is higher than the theoretical specific capacity of Co_3_O_4_.

In summary, porous graphitic carbon nanosheets encapsulating small and uniform Co_3_O_4_ NPs with an average grain diameter of about 20.4 nm were successfully fabricated. In this strategy, Co_3_O_4_@C@PGC nanosheets were compounded using glucose as the carbon source, cobalt nitrate as the Co_3_O_4_ precursor, and a surface of sodium chloride as the template. In this unique architecture, the PGC nanosheets with high elasticity can effectively preserve the structural and interfacial stabilization of Co_3_O_4_ NPs as well as accommodate the mechanical stress resulting from the severe volume change of Co_3_O_4_ NPs during lithium ion insertion/extraction, while the interconnected porous graphitic carbon networks with large surface area, superior electrical conductivity, high mechanical flexibility and stability lead to remarkably enhanced structural and electrical integrity as well as excellent kinetics for ion and electron transport of the overall electrode. As a result, such a nanostructured electrode exhibits an extremely durable high-rate capability: a capacity of 1030 mA h g^−1^ is achieved at 2 C, 560 mA h g^−1^ at 10 C, and 345 mA h g^−1^ at 20 C. Cycling at 5 C after 1000 cycles leads to a recovered capacity of 760 mA h g^−1^, still almost 2 times more than the capacity of graphite. The obtained good performance opens up new opportunities in the development of high performance next-generation LIB used for alternative energy and electric transportation.

## Methods

### Synthesis of Co_3_O_4_@C@PGC nanosheets

All the chemicals were of analytical grade and were used without further purification. Firstly, 1.8 g of glucose (Sinopharm Chemical Reagent Co., LTD. AR), 0.582 g of Co(NO_3_)_2_.6H_2_O(Sinopharm Chemical Reagent Co., LTD. AR) and 15 g of NaCl (Tianjin Guangfu Technology Development Co., LTD. AR)were dissolved in 50 ml of deionized water under magnetic stirring until a transparent blue solution was obtained. Then, the solution was transfered into a drying oven and dried at 80 ^o^C for 24 h. The dry block of crystal was ground to fine powder in an agatemortar. The composite powder was heated in a tubular vacuum furnace at 750 ^o^C for 2 h under flowing N_2_. Finally, it was annealed again at 300 ^o^C for 4 h under air. Once cooled to room temperature, the as-synthesized black powder was washed by deionized water several times to remove NaCl, and then Co_3_O_4_@C@PGC nanosheets were obtained. For comparison, Co_3_O_4_/C composites were also synthesized by carbonizing the mixture of Co(NO_3_)_2_·6H_2_O and glucose without adding NaCl at the same conditions. Meanwhile, Co_3_O_4_ NPs were also synthesized by sintering Co_3_O_4_@C@PGC nanosheets at 600 ^o^C for 2 h in air. And PGC nanosheets were obtained by immersing Co_3_O_4_@C@PGC nanosheets in plenty of nitric acid for 4 h at 70 ^o^C with magnetic stirring to remove the Co_3_O_4_ NPs.

### Materials Characterization

XRD measurements were taken on X-ray diffractometer (XRD, Rigaku D/max) with Cu Kα radiation at a wavelength of 1.5406 Å to determine the phase composition and crystallinity. Field emission scanning electron microscope (FESEM) images were acquired on a JEOL JSM-6700F micro-scope operated at 5 kV. The transmission electron microscope (TEM), high-resolution TEM (HR-TEM) images were recorded on a JEOL JEM-2100F high-resolution transmission electron microscope at 200 kV. Thermogravimentry analysis (TGA) was performed with a Perkin-Elmer (TA Instruments) instrument up to 750 ^o^C at a heating rate of 10 ^o^C min^−1^ in air. The Raman spectrum was recorded on the Lab RAM HR Raman spectrometer using laser excitation at 514.5 nm from an argon ion laser source to validate the Co_3_O_4_@C@PGC nanosheets. Brunauer-Emmett-Teller (BET) surface areas and porosities of the products were determined by nitrogen adsorption and desorption using a Micromeritics ASAP 2020 analyzer.

### Electrochemical Measurements

Electrochemical measurements were conducted on a coin-type test cell (CR2025) with metallic lithium sheet as both counter and reference electrode. The working electrode is fabricated by mixing active material (Co_3_O_4_@C@PGC nanosheets, Co_3_O_4_/C composites, Co_3_O_4_ NPs and PGC nanosheets), conductive material (acetylene black, Hong-xin Chemical Works) and binder polyvinylidene fluoride (PVDF, DuPont Company, 99.9%) in a weight ratio of 8:1:1 with N-methyl-2-pyrrolidone (NMP, Aladdin Reagent, AR) as solvent. The mixture was fully stirred for several hours in a weighing bottle into slurry. Then, it was uniformly pasted on Cu foil substrates. After vacuum drying at 120 ^o^C for 12 h, the electrode disks (*d* = 12 mm) were punched and weighed. The working electrode has approximately 0.5–1.0 mg of active material. The diameter of counter electrode (16 mm) is larger than working electrode. The electrolyte is 1 M LiPF6 in a mixture of ethylene carbonate and diethyl carbonate (1:1 by weight) and a Celgard 2400 polypropylene membrane was used as the separator. The cells were assembled in an argon-filled glove box in which both the moisture and oxygen contents were controlled to be less than 0.1 ppm. Cyclic voltammetry (CV) was performed on an IVIUMSTAT electrochemical workstation at 0.1 mV s^−1^ in the range of 0.01−3.0 V. Galvanostatic charge-discharge cycling tests were performed using an LAND CT2001A battery testing system in the voltage range between 0.01 V and 3 V. Electrochemical impedance spectroscopy (EIS) experiments were carried out using an IVIUMSTAT electrochemical workstation in the frequency range of 0.01−100 kHz at room temperature. All of the specific capacities in this work were estimated by using the weight of the active materials.

## Additional Information

**How to cite this article**: Leng, X. *et al.* Carbon-Encapsulated Co_3_O_4_ Nanoparticles as Anode Materials with Super Lithium Storage Performance. *Sci. Rep.* **5**, 16629; doi: 10.1038/srep16629 (2015).

## Supplementary Material

Supplementary Information

## References

[b1] Armand M. & Tarascon J. Building better batteries. Nature 451, 652–657 (2008). 18256660 10.1038/451652a

[b2] Tollefson J. Car industry: charging up future. Nature 456, 436–440 (2008). 19037290 10.1038/456436a

[b3] Kim H., Seo D., Kim S., Kim J. & Kang K. Highly reversible Co_3_O_4_/graphene hybrid anode for lithium rechargeable batteries. Carbon 49, 326–332 (2011).

[b4] Ji L., Lin Z., Alcoutlabi M. & Zhang X. Recent developments in nanostructured anode materials for rechargeable lithium-ion batteries. Energy Environ. Sci. 4, 2682 (2011).

[b5] Guo J., Chen L., Zhang X., Jiang B. & Ma L. Sol-gel synthesis of mesoporous Co_3_O_4_ octahedra toward high-performance anodes for lithium-ion batteries. Electrochim. Acta 129, 410–415 (2014).

[b6] Huang X. *et al.* Homogeneous CoO on Graphene for Binder-Free and Ultralong-Life Lithium Ion Batteries. Adv. Funct. Mater. 23, 4345–4353 (2013).

[b7] Huang Y. *et al.* Self-assembly of ultrathin porous NiO nanosheets/graphene hierarchical structure for high-capacity and high-rate lithium storage. J. Mater. Chem. 22, 2844–2847 (2012).

[b8] Kang E. *et al.* Fe_3_O_4_ nanoparticles confined in mesocellular carbon foam for high performance anode materials for lithium-ion batteries. Adv. Funct. Mater. 21, 2430–2438 (2011).

[b9] Li A., Song H., Wan W., Zhou J. & Chen X. Copper oxide nanowire arrays synthesized by *in-situ* thermal oxidation as an anode material for lithium-ion batteries. Electrochim. Acta 132, 42–48 (2014).

[b10] Song J., Duay J., Gillette E. & Lee S. The reversible anomalous high lithium capacity of MnO_2_ nanowires. Chem. Commun. 50, 7352–7355 (2014). 10.1039/c4cc02001d 24871137

[b11] Zhang M. *et al.* Flexible CoO–graphene–carbon nanofiber mats as binder-free anodes for lithium-ion batteries with superior rate capacity and cyclic stability. J. Mater. Chem. A 2, 5890 (2014).

[b12] Liu J. *et al.* Multifunctional CoO@C metasequoia arrays for enhanced lithium storage. Nano Energy 7, 52–62 (2014).

[b13] Hou C. *et al.* Integrated solid/nanoporous copper/oxide hybrid bulk electrodes for high-performance lithium-ion batteries. Sci. Rep. 3, 2878 (2013). 24096928 10.1038/srep02878 PMC3791456

[b14] Poizot P., Laruelle S., Grugeon S., Dupont L. & Tarascon J. Nano-sized transition-metal oxides as negative-electrode materials for lithium-ion batteries. Nature 407, 496–499 (2000). 11028997 10.1038/35035045

[b15] Ma J. *et al.* A solvothermal strategy: one-step *in situ* synthesis of self-assembled 3D graphene-based composites with enhanced lithium storage capacity. J. Mater. Chem. A 2, 9200 (2014).

[b16] Zhan L., Wang Y., Qiao W., Ling L. & Yang S. Hollow carbon spheres with encapsulation of Co_3_O_4_ nanoparticles as anode material for lithium ion batteries. Electrochim. Acta 78, 440–445 (2012).

[b17] Poizot P., Laruelle S., Grugeon S., Dupont L. & Tarascon J. Nano-sized transition-metal oxides as negative-electrode materials for lithium-ion batteries. Nature 407, 496–499 (2000). 11028997 10.1038/35035045

[b18] Jiang C., Hosono E. & Zhou H. Nanomaterials for lithium ion batteries. Nano Today 1, 28–33 (2006).

[b19] Guo B., Li C. & Yuan Z. Nanostructured Co_3_O_4_ materials: synthesis, characterization, and electrochemical behaviors as anode reactants in rechargeable lithium ion batteries. J. Phys. Chem. C 114, 12805–12817 (2010).

[b20] Li Y., Tan B. & Wu Y. Mesoporous Co_3_O_4_ nanowire arrays for lithium ion batteries with high capacity and rate capability. Nano lett. 8, 265–270 (2008). 18072799 10.1021/nl0725906

[b21] Li L. *et al.* The facile synthesis of hierarchical porous flower-like NiCo_2_O_4_ with superior lithium storage properties. J. Mater. Chem. A 1, 10935 (2013).

[b22] Wang X. *et al.* Mesoporous NiO nanosheet networks as high performance anodes for Li ion batteries. J. Mater. Chem. A 1, 4173 (2013).

[b23] Li L. *et al.* Co_3_O_4_ mesoporous nanostructures@graphene membrane as an integrated anode for long-life lithium-ion batteries. J. Power Sources 255, 52–58 (2014).

[b24] Wang R., Xu C., Sun J., Gao L. & Lin C. Flexible free-standing hollow Fe_3_O_4_/graphene hybrid films for lithium-ion batteries. J Mater. Chem. A 1, 1794 (2013).

[b25] Choi B. *et al.* 3D heterostructured architectures of Co_3_O_4_ nanoparticles deposited on porous graphene surfaces for high performance of lithium ion batteries. Nanoscale 4, 5924–5930 (2012). 22899185 10.1039/c2nr31438j

[b26] Wang L. *et al.* Nitrogen-doped porous carbon/Co_3_O_4_ nanocomposites as anode materials for lithium-ion batteries. ACS Appl. Mater. Interfaces 6, 7117–7125 (2014). 24802130 10.1021/am406053s

[b27] Nam K. *et al.* Virus-enabled synthesis and assembly of nanowires for lithium ion battery electrodes. Science 312, 885–888 (2006). 16601154 10.1126/science.1122716

[b28] Jayaprakash N., Jones W., Moganty S. & Archer L. Composite lithium battery anodes based on carbon@Co_3_O_4_ nanostructures: synthesis and characterization. J. Power Sources 200, 53–58 (2012).

[b29] Wang Y. *et al.* Designed functional systems from peapod-like Co@carbon to Co_3_O_4_@carbon nanocomposites. Acs Nano 4, 4753–4761 (2010). 20666372 10.1021/nn1004183

[b30] Hao F., Zhang Z. & Yin L. Co_3_O_4_/carbon aerogel hybrids as anode materials for lithium-ion batteries with enhanced electrochemical properties. ACS Appl. Mater. Interfaces 5, 8337–8344 (2013). 23924311 10.1021/am400952j

[b31] Wu Z. *et al.* Graphene Anchored with Co_3_O_4_ nanoparticles as anode of lithium ion batteries with enhanced reversible capacity and cyclic performance. ACS Nano 4, 3187–3194 (2010). 20455594 10.1021/nn100740x

[b32] Zhuo L. *et al.* Facile synthesis of a Co_3_O_4_–carbon nanotube composite and its superior performance as an anode material for Li-ion batteries. J. Mater. Chem. A 1, 1141 (2013).

[b33] Sun H. *et al.* Graphene-wrapped mesoporous cobalt oxide hollow spheres anode for high-rate and long-life lithium ion batteries. J. Phys. Chem. C 118, 2263–2272 (2014).

[b34] Gu D. *et al.* Controllable synthesis of mesoporous peapod-like Co_3_O_4_@carbon nanotube arrays for high-performance lithium-ion batteries. Angew. Chem. Int. Ed. 54, 7060–7064 (2015). 10.1002/anie.201501475 25914341

[b35] Peng L., Feng Y., Bai Y., Qiu H. & Wang Y. Designed synthesis of hollow Co_3_O_4_ nanoparticles encapsulated in a thin carbon nanosheet array for high and reversible lithium storage. J. Mater. Chem. A 3, 8825–8831 (2015).

[b36] Guo H., Sun Q., Li X., Wang Z. & Peng W. Synthesis and electrochemical performance of Co_3_O_4_/C composite anode for lithium ion batteries. Trans. Nonferrous Met. Soc. China 19, 372–376 (2009).

[b37] Qin J. *et al.* Graphene networks anchored with Sn@graphene as lithium ion battery anode. ACS Nano 8, 1728–1738 (2014). 24400945 10.1021/nn406105n

[b38] Chakraborty D. & Patey G. Evidence that crystal nucleation in aqueous NaCl solution occurs by the two-step mechanism. Chem. Phys. Lett. 587, 25–29 (2013).

[b39] Chakraborty D. & Patey G. How crystals nucleate and grow in aqueous NaCl solution. J. Phys. Chem. Lett. 4, 573–578 (2013). 26281868 10.1021/jz302065w

[b40] Yuvaraj S., Lin F., Chang T. & Yeh C. Thermal decomposition of metal nitrates in air and hydrogen environments. J. Phys. Chem. B 107, 1044–1047 (2003).

[b41] Wang D., Li F., Liu M., Lu G. & Cheng H. 3D aperiodic hierarchical porous graphitic carbon material for high-rate electrochemical capacitive energy storage. Angew. Chem. Int. Ed. 47, 373–376 (2008). 10.1002/anie.200702721 18022983

[b42] Huang C., Doong R., Gu D. & Zhao D. Dual-template synthesis of magnetically-separable hierarchically-ordered porous carbons by catalytic graphitization. Carbon 49, 3055–3064 (2011).

[b43] Wen F. *et al.* Fabrication of carbon encapsulated Co_3_O_4_ nanoparticles embedded in porous graphitic carbon nanosheets for microwave absorber. Carbon 89, 372–377 (2015).

[b44] Yang Z., Shen J. & Archer L. An *in situ* method of creating metal oxide–carbon composites and their application as anode materials for lithium-ion batteries. J. Mater. Chem. 21, 11092 (2011).

[b45] Wang. K. *et al.* N-doped carbon encapsulated Co_3_O_4_ nanoparticles as a synergistic catalyst for oxygen reduction reaction in acidic media. Int. J. Hydrogen Energ. 40, 3875–3882 (2015).

[b46] Zhang W., Wu X., Hu J., Guo Y. & Wan L. Carbon coated Fe_3_O_4_ nanospindles as a superior anode material for lithium-ion batteries. Adv. Funct. Mater. 18, 3941–3946 (2008).

[b47] Zhu T., Chen J. & Lou X. Glucose-assisted one-pot synthesis of FeOOH nanorods and their transformation to Fe_3_O_4_@carbon nanorods for application in lithium ion batteries. J. Phys. Chem. C 115, 9814–9820 (2011).

[b48] Luo B. *et al.* Reduced graphene oxide-mediated growth of uniform tin-core/carbon-sheath coaxial nanocables with enhanced lithium ion storage properties. Adv. Mater. 24, 1405–1409 (2012). 22302438 10.1002/adma.201104362

[b49] Wang B. *et al.* Adaptable silicon–carbon nanocables sandwiched between reduced graphene oxide sheets as lithium ion battery snodes. ACS Nano 7, 1437–1445 (2013). 23281801 10.1021/nn3052023

[b50] Xiong S., Chen J., Lou X. & Zeng H. Mesoporous Co_3_O_4_ and CoO@C topotactically transformed from chrysanthemum-like Co(CO_3_)_0.5_(OH)·0.11H_2_O and their lithium-storage properties. Adv. Funct. Mater. 22, 861–871 (2012).

[b51] Chen Y. *et al.* Reduced graphene oxide networks as an effective buffer matrix to improve the electrode performance of porous NiCo_2_O_4_ nanoplates for lithium-ion batteries. J. Mater. Chem. A 2, 4449 (2014).

[b52] Jia X. *et al.* Building robust architectures of carbon and metal oxide nanocrystals toward high-performance anodes for lithium-ion batteries. ACS Nano 6, 9911–9919 (2012). 23046380 10.1021/nn303478e

[b53] Liang C. *et al.* The structure dependent electrochemical performance of porous Co_3_O_4_ nanoplates as anode materials for lithium-ion batteries. J. Power Sources 251, 351–356 (2014).

[b54] Wang J. *et al.* Accurate control of multishelled Co_3_O_4_ hollow microspheres as high-performance anode materials in lithium-ion batteries. Angew. Chem., Int. Ed. 52, 6417–6420 (2013). 10.1002/anie.201301622 23649876

[b55] Yang S., Feng X., Ivanovici S. & Mullen K. Fabrication of graphene-encapsulated oxide nanoparticles: towards high-performance anode materials for lithium storage. Angew. Chem., Int. Ed. 49, 8408–8411 (2010). 10.1002/anie.201003485 20836109

[b56] Peng C. *et al.* Facile ultrasonic synthesis of CoO quantum dot_graphene nanosheet composites with high lithium storage capacity. ACS Nano 6, 1074–1081 (2012). 22224549 10.1021/nn202888d

[b57] Wang R. *et al.* Free-standing and binder-free lithium-ion electrodes based on robust layered assembly of graphene and Co_3_O_4_ nanosheets. Nanoscale 5, 6960–6967 (2013). 23793785 10.1039/c3nr01392h

[b58] Poizot P., Laruelle S., Grugeon S. & Tarascon J. Nano-sized transition-metal oxides as negative-electrode materials for lithium-ion batteries. Nature 407, 496–499 (2000). 11028997 10.1038/35035045

[b59] He C. *et al.* Carbon-encapsulated Fe_3_O_4_ nanoparticles as a high-rate lithiumion battery anode material. ACS Nano 7, 4459–4469 (2013). 23614734 10.1021/nn401059h

[b60] Zhu J. *et al.* Topochemical transformation route to atomically thick Co_3_O_4_ nanosheets realizing enhanced lithium storage performance. Nanoscale 5, 5241–5246 (2013). 23649213 10.1039/c3nr01178j

[b61] Simon P. & Gogotsi Y. Materials for electrochemical capacitors. Nat. Mater. 7, 845–854 (2008). 18956000 10.1038/nmat2297

[b62] Chen L. *et al.* Porous graphitic carbon nanosheets as a high-rate anode material for lithium-ion batteries. ACS Appl. Mater. Interfaces 5, 9537–9545 (2013). 24016841 10.1021/am402368p

[b63] Fang Y. *et al.* Two-dimensional mesoporous carbon nanosheets and their derived graphene nanosheets: synthesis and efficient lithium ion storage. J. Am. Chem. Soc. 135, 1524–1530 (2013). 23282081 10.1021/ja310849c

[b64] Li B. *et al.* Co_3_O_4_@graphene composites as anode materials for high-performance lithium ion batteries. Inorg. Chem. 50, 1628–1632 (2011). 21244033 10.1021/ic1023086

[b65] Mo Y., Ru Q., Song X., Hu S. & An B. A novel dendritic crystal Co_3_O_4_ as high-performance anode materials for lithium-ion batteries. J. Appl. Electrochem. 44, 781–788 (2014).

[b66] Xing L., Chen Z. & Xue X. Controllable synthesis Co_3_O_4_ nanorods and nanobelts and their excellent lithium storage performance. Solid State Sci. 32, 88–93 (2014).

[b67] Yang X. *et al.* Electric papers of graphene-coated Co_3_O_4_ fibers for high-performance lithium-ion batteries. ACS Appl. Mater. Interfaces 5, 997–1002 (2013). 23320959 10.1021/am302685t

[b68] Zheng J. & Zhang B. Facile chemical bath deposition of Co_3_O_4_ nanowires on nickel foam directly as conductive agent- and binder-free anode for lithium ion batteries. Ceram. Int. 40, 11377–11380 (2014).

[b69] Huang G., Xu S., Lu S., Li L. & Sun & H. Micro-/nanostructured Co_3_O_4_ anode with enhanced rate capability for lithium-ion batteries. ACS Appl. Mater. Interfaces 6, 7236–7243 (2014). 24791835 10.1021/am500452t

[b70] Chen S. & Wang Y. Microwave-assisted synthesis of a Co_3_O_4_–graphene sheet-on-sheet nanocomposite as a superior anode material for Li-ion batteries. J. Mater. Chem. 20, 9735 (2010).

