# Supplementary material for: Carbon-Encapsulated Co3O4 Nanoparticles as Anode Materials with Super Lithium Storage Performance
Source: Sci Rep. 2015 Nov 13;5:16629. doi: 10.1038/srep16629 (PMC4643224; doi:10.1038/srep16629)
Supplement: Supplementary Information [file srep16629-s1.pdf]

Supplementary Information

for

Carbon-Encapsulated Co_3O_4 Nanoparticles as Anode Materials with Super Lithium Storage Performance

Xuning Leng[1], Sufeng Wei[2], Zhonghao Jiang[1], Jianshe Lian[1], Guoyong Wang[1,*], Qing Jiang[1]

[1]Key Laboratory of Automobile Materials, Department of Materials Science and Engineering, Jilin University, No. 5988 Renmin Street, Changchun 130025, PR China

[2]Key Laboratory of Advanced Structural Materials, Changchun University of Technology, Changchun 130012, PR China

*Corresponding author: Tel.: +86 431 85095875; Fax: +86 431 85095876

E-mail address: materwanggy@jlu.edu.cn

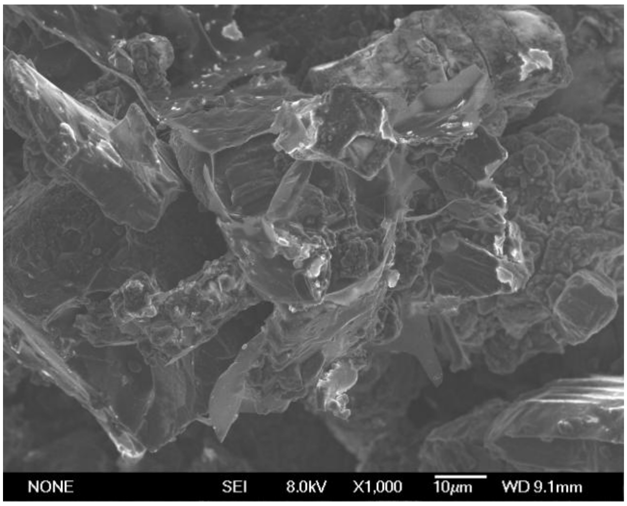

Figure S1. SEM image of the Co_3O_4@C@PGC nanosheets coated NaCl after two-step heat treatment.

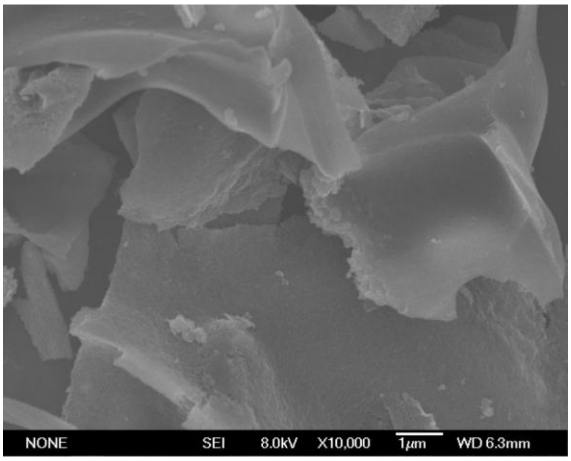

Figure S2. SEM image of the carbonization products of the mixture ($Co(NO_3)_2$ $6H_2O$ and $C_6H_{12}O_6$) without adding NaCl.

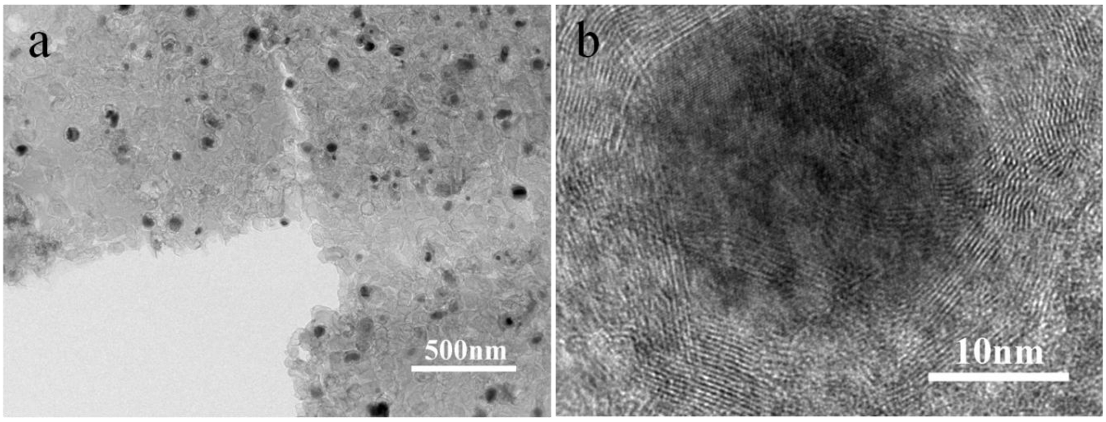

Figure S3 (a) TEM and (b) HRTEM images of Co@C@PGC nanosheets.

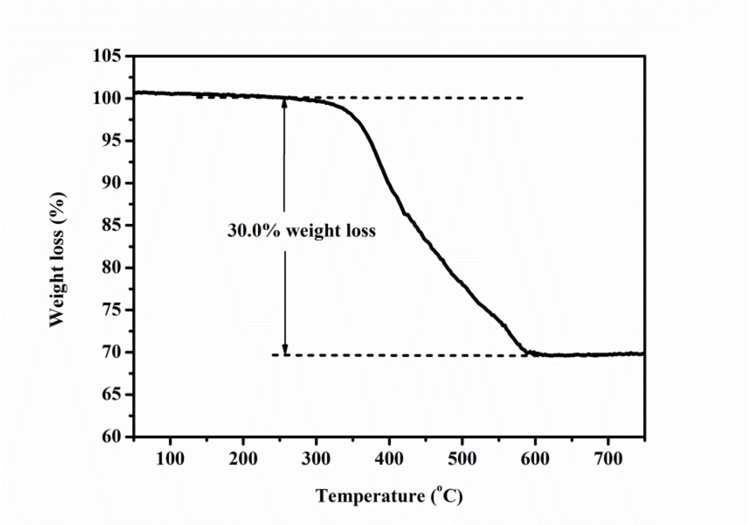

Figure S4. The TGA profile of the Co_3O_4/C composite.

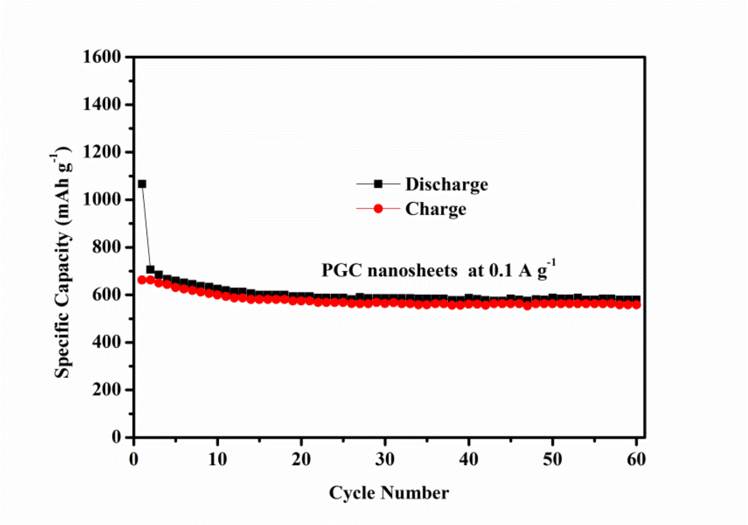

Figure S5. Charge/discharge capacities of the PGC nanosheets at a current density of 0.1 C.

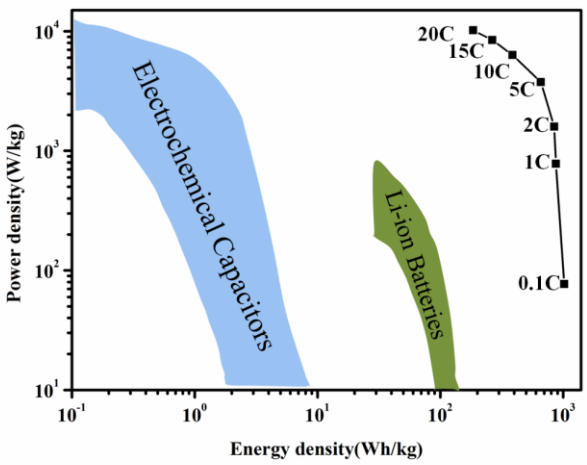

Figure S6. The power density and energy density of the Co_3O_4@C@PGC nanosheets electrode are adding in the Ragone plot and compared electrochemical capacitors and traditional LIBs. The high reversible capacity and excellent rate performance of the electrode, in fact, unite the advantage of electrochemical capacitors and traditional LIBs. So it occupies the up-right corner of the Ragone plot.

Table S1. Comparison of synthesis techniques and structures from reported literatures.

synthetic technique	structure or morphology	references
in situ chemical reduction process, followed by a heat treatment	porous Co_3O_4/graphene thin sheet hybrid structure	Ref. 3
chemical deposition of Co_3O_4 NPs onto graphene oxide (GO), followed by reduction of GO to graphene in a $NaBH_4$ solution	Co_3O_4 NPs@graphene composite	Ref. 64
homogeneous co-precipitation method in the presence of oxalic as complex agent, followed by a heat treatment	dendritic structure composed of Co_3O_4 NPs with a size of 20–50 nm	Ref. 65
template-free hydrothermal method, followed by a heat treatment	Co_3O_4 nanobelts constructed with a large amount of nanorods with porous structure	Ref. 66
bonding between glucose molecules and $Co(CO_3)_{0.5}(OH)0.11H_2O$ under hydrothermal conditions, following calcinations in Ar and annealing in air in sequence	peapod-like Co_3O_4@carbon structure	Ref. 29
Co_3O_4 fibers were prepared by electrospinning technique, then combining GO by electrostatic interactions, followed by filtration	Electric Papers of Graphene-Coated Co_3O_4 Fibers	Ref. 67
facile chemical bath deposition route under a mild condition	Co_3O_4 nanowires on nickel foam	Ref. 68
the growth of Co_3O_4 NPs on the surface of f-CNTs by thermal decomposition of cobalt nitrate hexahydrate in ethanol	Co_3O_4 particles grew compactly and uniformly along the carbon nanotube axis	Ref. 32
combine β-$Co(OH)_2$ sheets with GO under sonication, then vacuum filtration and thermal treatment	robust layered assembly of graphene and Co_3O_4 nanosheets	Ref. 57
firstly obtain $Co(OH)_2$/graphene composite precursor, then calcination	Co_3O_4 NPs anchored on conducting graphene	Ref. 31
Firstly prepare hollow and mesoporous Co_3O_4 spheres by a hydrothermal method, then combine with graphene by a three-step fabrication procedure	Graphene-wrapped mesoporous Co_3O_4 hollow spheres	Ref. 33
hydrothermal method with a special surfactant triethanolamine followed by	monodispersed porous Co_3O_4 cubes with	Ref. 69

thermal treatment	micro-/nanostructure	
microwave-assisted synthesis of graphene oxide/Co(OH)$_2$ composite, followed by thermal treatment	Co_3O_4/graphene sheet-on-sheet nanostructure	Ref. 70
sol-gel technology with triblock copolymer F127 as the soft template	nanosized porous Co_3O_4 octahedra	Ref. 5
hexagon single-crystal Co(OH)$_2$ nanoplates obtained by hydrothermal synthesis method, then transformed to the porous single-crystal Co_3O_4 nanoplates by heat treatment	porous single-crystal Co_3O_4 nanoplates	Ref. 53
preparation by using glucose as the carbon source, cobalt nitrate as the Co_3O_4 precursor, and a surface of sodium chloride as the template, followed by two-step heat treatment	porous Co_3O_4@C@PGC nanosheets	This work

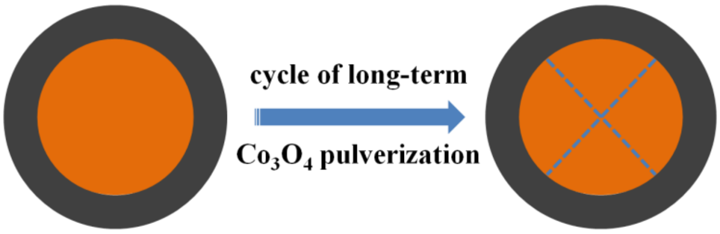

Figure S7. Schematic illustration of the Co_3O_4 core embedded in carbon shell with the probable pulverization after a cycle of long-term.
